# Cloning of Interleukin-10 from African Clawed Frog (*Xenopus tropicalis*), with the Finding of IL-19/20 Homologue in the IL-10 Locus

**DOI:** 10.1155/2015/462138

**Published:** 2015-02-10

**Authors:** Zhitao Qi, Qihuan Zhang, Zisheng Wang, Weihong Zhao, Qian Gao

**Affiliations:** ^1^Key Laboratory of Aquaculture and Ecology of Coastal Pool of Jiangsu Province, Department of Ocean Technology, Yancheng Institute of Technology, Yancheng, Jiangsu 224051, China; ^2^Central Laboratory of Biology, Chemical and Biological Engineering College, Yancheng Institute of Technology, Yancheng, Jiangsu 224051, China; ^3^State Key Laboratory of Freshwater Ecology and Biotechnology, Institute of Hydrobiology, Chinese Academy of Sciences, Wuhan, Hubei 430070, China

## Abstract

Interleukin-10 (IL-10) is a pleiotropic cytokine that plays an important role in immune system. In the present study, the IL-10 gene of African clawed frog (*Xenopus tropicalis*) was first cloned, and its expression pattern and 3D structure were also analyzed. The frog IL-10 mRNA encoded 172 amino acids which possessed several conserved features found in IL-10s from other species, including five-exon/four-intron genomic structure, conserved four cysteine residues, IL-10 family motif, and six *α*-helices. Real-time PCR showed that frog IL-10 mRNA was ubiquitous expressed in all examined tissues, highly in some immune related tissues including kidney, spleen, and intestine and lowly in heart, stomach, and liver. The frog IL-10 mRNA was upregulated at 24 h after LPS stimulation, indicating that it plays a part in the host immune response to bacterial infection. Another IL, termed as IL-20, was identified from the frog IL-10 locus, which might be the homologue of mammalian IL-19/20 according to the analysis results of the phylogenetic tree and the sequence identities.

## 1. Introduction

Based on structural features, the cytokines have been grouped into several families such as interleukin (IL), interferon (IFN), tumor necrosis factor (TNF), and transforming growth factor (TGF) family [1]. The homologies among genes from different families are quite limited [2]. According to sequence homologies, similarities of receptor chain, and functional properties, ILs are further divided into IL-1, IL-10, IL-12, and IL-17 families [3]. IL-10 family consists of nine members: IL-10, IL-19, IL-20, IL-22, IL-24, IL-26, IL28A, IL-28B, and IL-29. In mammals these members clustered, respectively, in three genomic loci: IL-10 locus in which IL-10, IL-19, IL-20, and IL-24 are included; IFN-*γ* locus in which IL-22 and IL-26 are included; IFN-*λ* locus in which IL-28A, IL-28B, and IL-29 (also called IFN-*λ*2, IFN-*λ*3, and IFN-*λ*1, resp.) are included [4]. These IL-10 family members in mammals are involved in diverse immune regulation and host defense during bacterial and viral infection and also were important for the differentiation and proliferation of immune cells, such as T cell, B cell, and natural killer (NK) cell [5].

Among these IL-10 family members, IL-10 was originally called cytokine synthesis inhibitory factor (CSIF), which was firstly cloned from Th2 clones, because of its inhibition on the production of several cytokines, such as IFN-*γ* [6]. Subsequently, it was renamed as IL-10 and found to be expressed by the cells involved in innate and adaptive immunity, including NK cells, dendritic cells (DCs), macrophages, mast cells, neutrophils, CD4+ and CD8+ T cells, and B cells [7, 8]. Several immunostimulants, such as LPS, polyI:C [9], phytohaemagglutinin (PHA), and phorbol 12-myristate 13-acetate (PMA) [10], could enhance the expression of IL-10 in the above-mentioned cells.

To data, several IL-10 family members have been identified from lower vertebrates. In fish IL-10 and IL-20L clustered in the IL-10 locus. The latter might be the ancestral that gave rise to IL-19, IL-20, and IL-24 genes [11]. Fish IL-22 and IL-26 genes were in IFN-*γ* locus, being similar to those in mammalian [12]. In amphibian, two IL-10 loci, that is, IFN-*γ* locus and IFN-*λ* locus, have been well characterized, with IFN-*γ* locus containing IL-22 and IL-26 genes [13] and IFN-*λ* locus IFN*λ*1-5 genes [14]. However, information about the IL-10 locus in amphibian was still blank.

Functional study in fish revealed that the IL-10 network in lower vertebrates was quite complex. For example, two trout IL-10s (IL-10a and IL-10b) possessed different expression pattern after stimuli stimulation and bacterial infection [15]. Goldfish IL-10 could downregulate the expression of the proinflammatory cytokines IL-1*β*, IL-8, and TNF-*α* and also IL-10 itself, indicating fish IL-10 might be an anti-inflammatory cytokine [16].

African clawed frog (*Xenopus tropicalis*) is the model animal of amphibian. Due to its special evolutionary position, it has long been used to gain better appreciation of the evolution of the complex immune system [17]. To provide more information on the evolution of IL-10 family, the frog IL-10 gene was cloned and structurally analyzed. Also, the interleukins in frog IL-10 locus were identified. And on this basis a comprehensive phylogenetic analysis was implemented in order to deepen the understanding of the evolution relationships among the various IL-10 family members in vertebrates.

## 2. Materials and Methods

### 2.1. *In Silico* Identification of Frog IL-10

To identify the frog IL-10 gene, human IL-10 and chicken IL-10 were used to query the genomic database of* X. tropicalis* (http://www.ensembl.org/) by tBLASTn program. The obtained genomic sequences were further analyzed using GenScan [18] and FGENESH+ [19] program to get the putative exon and intron boundary. The predictive genes were searched against the National Center for Biotechnology Information (NCBI) database (http://blast.ncbi.nlm.nih.gov) using BLASTp software. The gene synteny was analyzed using Genomicus (v75.02) software.

### 2.2. Cloning of Frog IL-10 cDNA

Total RNA was extracted from frogspleen using Trizol reagent (Invitrogen, USA) and transcripted into cDNA using Superscript II reverse transcription system (Invitrogen, USA) according to the manufacturer's instructions. The full cDNA sequence of frog IL-10 was obtained by using 3′- and 5′-RACE PCR method with the synthesized cDNA as template. The primers used for 3′- and 5′-RACE were designed based on the predicted results from GenScan and FGENESH+ analysis. The primers for 5′-RACE were UPM/IL10-5Rout (first round) and UPM/IL10-5Rin (second round) and UPM/IL10-3Fout (first round) and UPM/IL10-3Fin (second round) for 3′-RACE (Table 1). PCR was carried out in 25 *μ*L reaction system as follows: 125 *μ*M of each dNTP, 0.2 *μ*M of each primer, 2.5 *μ*L 10× Taq buffer, 12 U Ex Taq polymerase (TaKaRa, Japan), 18.1 *μ*L sterile H_2_O, and 1 *μ*L cDNA template according to the standard protocol. PCR amplification was conducted under the following conditions: an initial denaturation step at 94°C for 5 min, followed by 6 cycles of 30 s at 94°C, 30 s at 64°C, and 1 min at 72°C, 30 cycles of 30 s at 94°C, 30 s at 62°C, and 1 min at 72°C, and finally an extension step at 72°C for 10 min. The products of first round PCR were 1 : 100 diluted with water and then used as the template for the second round PCR. 10 *μ*L of second round PCR products was size-fractioned by 1.5% (w/v) agarose gel electrophoresis and stained with ethidium bromide. The desired PCR products were ligated into pMD18-T vectors (TaKaRa, Japan) and sequenced using the dideoxy chain termination method on an automatic DNA sequencer (ABI Applied Biosystems Mode 377).

### 2.3. Sequence Analyses

The deduced amino acid sequences were predicted using the Translate program. The molecular weight and the net charge of the protein were calculated by ProtParam program (http://ca.expasy.org/tools). The multiple protein sequence alignment was performed using CLUSTAL W program (version 1.83) [20]. Identities between the sequences were determined using Megalign program within DNASTAR software package. The neighbor-joining (N-J) phylogenetic tree was constructed using Jones-Taylor-Thornton (JTT) model within MEGA6 software [21]. The signal peptide and the N-glycosylation sites were predicted using SignalP (v2.0) [22] and NetGlyc 1.0 [23] server, respectively.

### 2.4. Modeling and Molecular Dynamics (MD) Simulation Analysis of Frog IL-10

The 3D structural model of frog IL-10 was constructed using comparative modeling method [24]. The template used for modeling was determined by searching in the GeneSilico Metaserver [25], pdbblast, and Pcons.net [26], with frog IL-10 amino acid sequence as “query.” Also, the template was confirmed by sequence-structure alignment using FUGUE (Find Homologs of Uncharacterized Gene Products Using Environment-specific substitution tables) program in the Homologous Structure Alignment Database (HOMSTARD), in which the target protein was clustered into homologous families and the top Z-score against the cut-off score (Z-score > 6.0) was considered the optimum template for modeling.

After validating the template for modeling, the models were generated by Swiss-PDB server (http://swissmodel.expasy.org/). Energy minimization of the obtained model was done in Swiss-PDB Viewer using a harmonic constraint of 100 kJ mol^−1^ Å^−2^ [27]. The quality of the model was checked by PROCHECK and ERRAT in SAVES server (http://nihserver.mbi.ucla.edu/). The model was displayed and analyzed with Swiss-PDB Viewer.

After obtaining the structural model of frog IL-10, explicit solvent MD simulation was performed using Gromacs (Groningen Machine for Chemical Simulations) 4.0 package [28] on an Inspur, 12 GHz PC equipped with the Red Hat 6.0 environment to further investigate the stability of this model. Briefly, the frog IL-10 protein was solvated by 17,297 water molecules in an octahedral box with 1.0 nm edges from the molecular boundary. Nine Cl^−^ ions were added to the frog IL-10 model with a net positive charge of +9 to obtain a neutral system. The configuration was energy minimized using the steepest descent algorithm (maximum number of steps: 4,000) to remove steric conflicts between the protein and water molecules. The energy-minimized models were stimulated for 100,000 steps for a total of 200 ps under 300 K using position-restrained MD in NPT conditions. Snapshots of the trajectory were taken every 1 ps. The final MD of 5,000,000 steps was carried out for 10,000 ps (10 ns) using the particle mesh Ewald (PME) electrostatics method under NPT conditions.

### 2.5. Animal

Healthy clawed frogs (*X. tropicalis*) were obtained from the Institute of Genetics and Developmental Biology, Chinese Academy of Sciences (Beijing, China), and maintained in a freshwater tank at 23°C under natural photoperiod and fed with pork liver twice per day. The animals were acclimatized for 1 week prior to experiments.

### 2.6. Tissue Distribution of Frog IL-10 mRNA

Tissue samples of heart, liver, kidney, spleen, stomach, and intestine were collected from three healthy frogs. The same tissues from three frogs were mixed together for RNA preparation using Trizol reagent and cDNA was synthesized with PrimeScript RT reagent kit (TaKaRa, Japan) according to the manufacturer's instructions. The cDNA fragments of frog IL-10 and *β*-actin were amplified by RT-PCR and confirmed by sequencing. Amplicons were gel purified, and serial tenfold dilutions were run along with the cDNA test samples on the same 96-well PCR plate as quantitative standard. The relative expression of frog IL-10 in various tissue samples was normalized to the expression of *β*-actin.

### 2.7. Modulation of the Expression of Frog IL-10 by LPS Stimulation

To characterize the change of frog IL-10 transcripts after LPS stimulation, three frogs were injected intraperitoneally (i.p.) with LPS (150 *μ*g/100 g body weight) and the frogs as control were injected with the same volume of PBS solution. Animals were anesthetized and killed at 24 h after injection. Then tissue collection, RNA extraction, and cDNA synthesis were conducted as described above. In the present study, the sampling time was determined according to our previous research [13]. The change of gene expression after LPS stimulation was expressed as fold change and calculated as described in our previous study [13, 14]. The data of real-time quantitative PCR were analyzed with the Origin 6.0 software. Results were expressed as mean values ± SD. A Student's* t*-test was applied to analyze the significance of fold change, with *P* value less than 0.05 considered as statistically significant difference.

## 3. Results

### 3.1. Sequence Analysis of Frog IL-10

**Figure 1:**
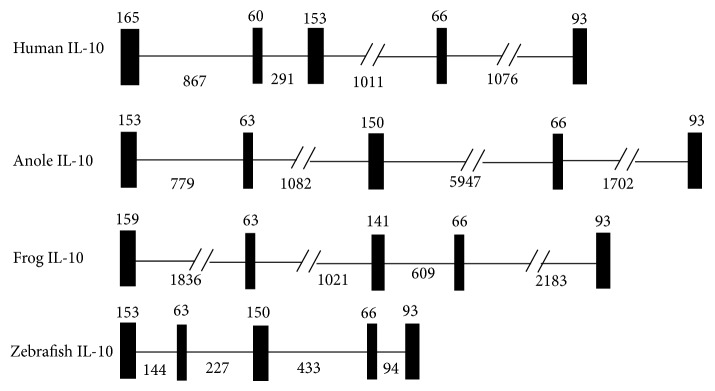
Comparison of the gene organization of frog IL-10 gene with selected IL-10 molecules. The exons were indicated as black boxes and introns as black lines. The numbers above each box indicate the size (bp) of exons and the numbers below the lines indicate the size (bp) of introns. The gene organization of each gene was extracted from Ensembl Genome database.

The full-length sequence of the frog IL-10 cDNA (Genbank accession number EF104912) comprised a 5′ terminal untranslated region (UTR) of 159 bp, an open reading frame (ORF) of 519 bp, and a 3′-UTR of 567 bp. No ATTTA sequence was observed in the 3′-UTR, which might involve in the shortening of the half-life of several cytokines and growth factors [29]. The genomic structure of frog IL-10 was composed of five exons and four introns. The size of the four introns was 1836 bp, 1021 bp, 609 bp, and 2183 bp, respectively, which was a little larger than the counterpart in human IL-10 or zebrafish IL-10. The exons of IL-10 genes were relatively conserved (Figure 1). The typical intron splice motifs, that is, GT and AG, were observed, respectively, at the 5′- and 3′-end of each intron.

**Figure 2:**
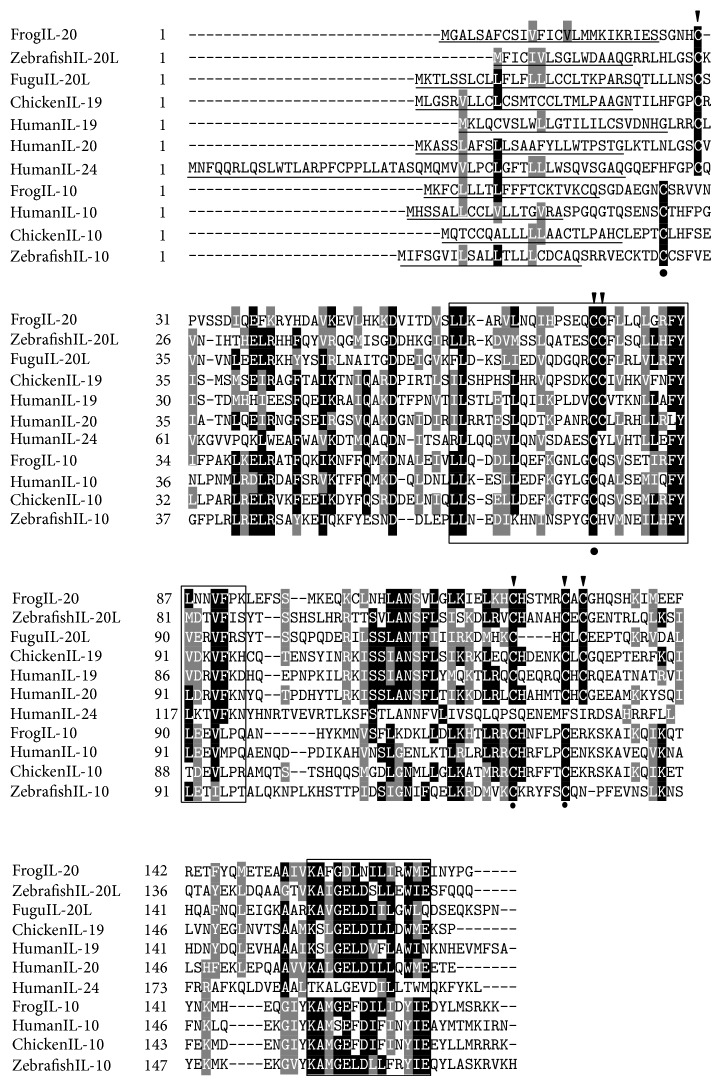
Multiple alignment of vertebrate IL-10. The multiple alignment was produced using Clustal W, and conserved amino acids were shaded using GeneDoc software. The signal peptides predicted by SignalP 4.1 server were underlined. The conserved IL-10 family signature motifs were boxed. The four conserved cysteine residues existing in IL-10 were indicated by black cycles below the alignment and the six conserved cysteine residues in IL-19/20 were indicated by black arrows above the alignment. The accession numbers for sequence used in this alignment were listed in Figure 6.

The putative protein of frog IL-10 was 172 amino acids in length, containing a 19 a.a. signal peptide at its N-terminus. The theoretical molecular weight and the isoelectric point (pI) of the mature peptide of frog IL-10 were 15.08 kDa and 7.92, respectively. Two conserved IL-10 family signature motifs, L-[FILMV]-X(3)-[ILV]-X(3)-[FILMV]-X(5)-C-X(5)-[ILMV]-[ILMV]-X(3)-L-X(2)-[IV]-[FILMV] and KA-X(2)-E-X-D-[ILV]-[FLY]-[FILMV]-X(2)-[ILMV]-[EKQZ], were found to manifest as LLQDDLLQEFKGNLGCQSVSETIRFYLEEVL and KAMGEFDILIDYIE in frog IL-10. In addition, four conserved cysteine residues were observed in frog IL-10. Two extra cysteine residues at the N-terminals of fish IL-10 [15] were not found in frog, bird, and mammalian IL-10s (Figure 2). The frog IL-10 shared the highest identity with chicken IL-10 (58.3%), followed by 56.5% with human IL-10 and 41.7% with zebrafish IL-10 (Table 2).

### 3.2. Modeling of Frog IL-10

By searching the GeneSilico Metaserver, pdbblast, and Pcons.net server, the human IL-10 crystal structure (PDB code: 2ILK) at 1.6 Å resolution was considered to be the optimal template for frogIL-10. This was also confirmed by a higher Z-score of 25.38 derived from the sequence-structure alignment between frogIL-10 and template using the FUGUE software. The high identity between a.a. sequences of frog IL-10 and template also confirmed that it was reasonable to do modeling using comparative modeling method.

The model of frog IL-10 was generated with Swill-PDB server and the quality of the resultant model was evaluated with PROCHECK and ERRAT software. The PROCHECK analysis showed that the phi-psi angles of 89.9% of the residues were in most favored regions, 7.1% in the additional allowed regions, 1.9% in generously allowed regions, and only 1.0% in disallowed regions. ERRAT program showed the overall quality factor of the frog IL-10 model was 96.31, which was more than 95%, indicating high resolution of structure. All these analyses suggested that this model could be used for further MD simulation.

**Figure 3:**
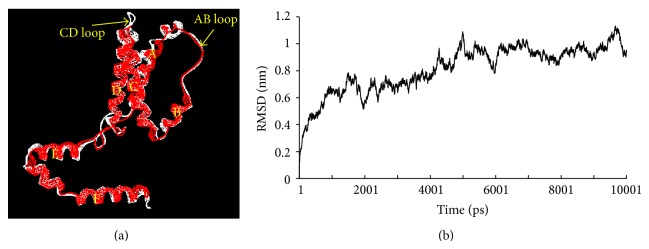
Structure and molecular dynamics analysis of frog IL-10. (a) Superimposition of the frog IL-10 model with the human IL-10 (PDB: 2ILK). Frog IL-10 was shown in red color and human IL-10 was in white color; (b) RMSD of frog IL-10 postmolecular dynamics for 12 ns. The MD analysis was performed by Gromacs 4.0 package [28] on an Inspur, 12 GHz PC, and results were analyzed using Origin 6.0 software.

The predicted spatial structure of frog IL-10 possessed six *α*-helices, similar to that of human IL-10 monomer. However, there were some slight differences between the two proteins; for example, helix A and helix C of frog IL-10 were a little shorter which results in a longer AB loop and CD loop (Figure 3(a)). MD analysis showed that the RMSD of frog IL-10 model became stable at 5 ns after simulation (Figure 3(b)). Mean RMSD and average RMSF (root mean square fluctuation) of frog IL-10 were 0.8146 ± 0.1744 nm and 0.3618 ± 0.2042 nm, respectively.

### 3.3. Expression Pattern of Frog IL-10

**Figure 4:**
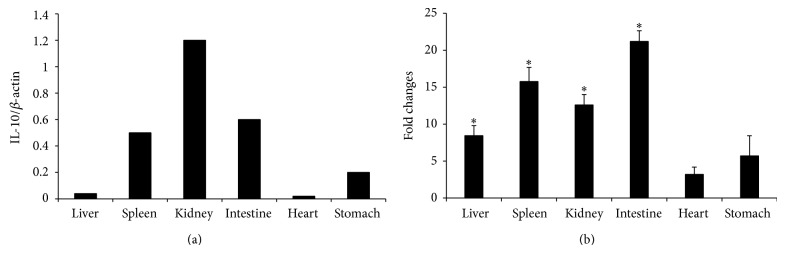
Expression analysis of frog IL-10 in different tissues from healthy frog (a) and LPS stimulated frog (b). Six tissues including liver, spleen, kidney, intestine, heart, and stomach were sampled for real-time PCR analysis. The mean ± SD values were shown. The transcripts levels of IL-10 in healthy frog were relative to those of *β*-actin. The expression changes of IL-10 in LPS stimulated frog were expressed as fold change relative to the controls. *P* values generated by paired sample *t*-test between control and stimulated groups were shown above the bars. ^*^ *P* < 0.05.

Real-time PCR was performed to examine the tissue expression pattern in healthy and LPS stimulated frogs. In healthy frog, IL-10 was highly expressed in kidney, moderately in spleen and intestine, and slightly in heart, liver, and stomach (Figure 4(a)).

After LPS stimulation for 24 h, the expression of frog IL-10 was apparently induced in liver, spleen, kidney, intestine, and stomach, showing 7.5-, 17.3-, 13.6-, 10.4-, and 5.3-fold changes, respectively (*P* < 0.05) (Figure 4(b)).

### 3.4. Gene Synteny Analysis of IL-10 Locus

**Figure 5:**
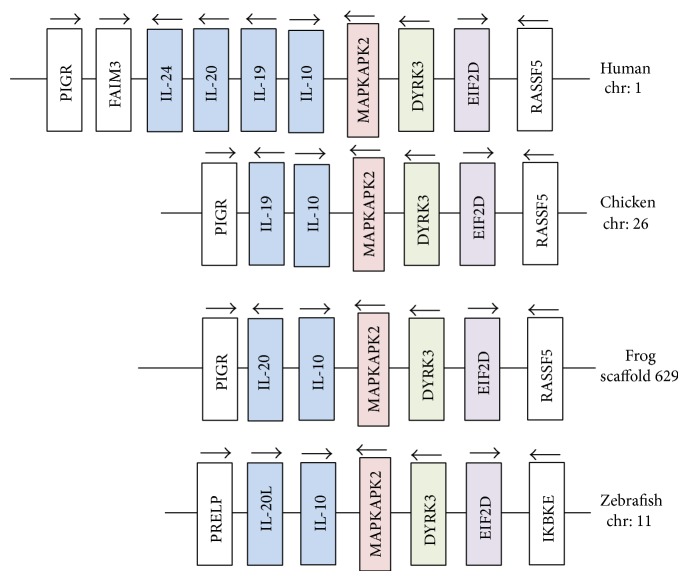
Gene synteny of IL-10 loci in vertebrates. The gene synteny of human, chicken, and zebrafish IL-10 locus was analyzed using Genomicus (v75.02) software and human IL-10 was set as a reference to compare the conserved synteny between different species. The gene synteny of frog IL-10 locus was based on the results of GenScan and BLAST search against NCBI database.

Based on* in silico* analysis, we totally identified two ILs from the frog IL-10 locus (Scaffold 629) which are named as IL-10 and IL-20. Gene synteny analysis showed that the IL-10 locus was well conserved during evolution. Many conserved genes, for example, MAPKAPK2, DYRK3, and EIF2D, highly linked with IL-10 locus were found in all analyzed species. There were four ILs (IL-10, -19, -20, and -24) in the human IL-10 locus and also IL-10 and -19 in chicken IL-10 locus, but without IL-20 and -24. Fish IL-10 locus also contained two ILs, merely being named as IL-10 and IL-20L [11] (Figure 5).

### 3.5. Phylogenetic Tree Analysis

**Figure 6:**
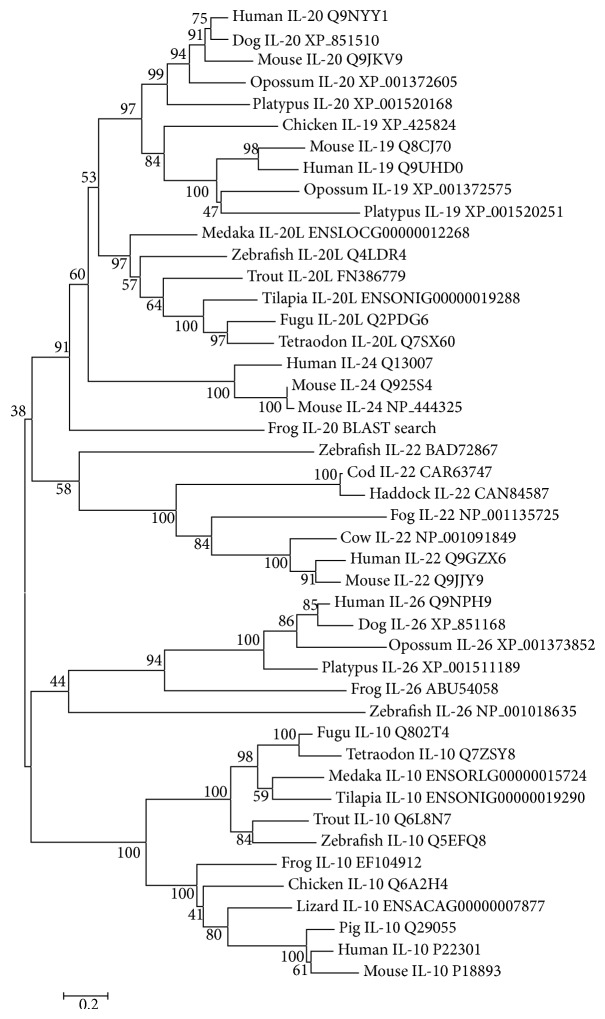
Phylogenetic tree analysis of IL-10 family members from frog and other species. The tree was constructed by the “neighbor-joining” method using MEGA 6.0 software. Node values represent the percent of bootstrap confidence derived from 1,000 replicates. The accession number for each sequence followed the common species name.

To further elucidate the evolution relationships of IL-10 family members, a phylogenetic tree was constructed using all the frog IL-10 family members except IFN-*λ*s and IL-10 family members from fish and higher vertebrates. It was clear that the IL-10 family members were mainly separated into four clusters with high bootstrap values, that is, IL-22, IL-26, IL-10, and IL19/20/24 clusters. Each of IL-22, IL-26, and IL-10 clusters contained all counterparts from fish, frog, and mammals. The IL19/20/24 cluster was further divided into three main clades: fish IL-20L clade, IL-19/20 clade, and mammal IL-24 clade. The IL-19/20 homologue newly identified from frog was grouped into the IL19/20/24 cluster and formed a sister group with all other cluster members (Figure 6).

## 4. Discussion

In the present study, the frog IL-10 gene was cloned, and its expression and 3D structure were also analyzed. The frog IL-10 has been found to share several conserved features with known IL-10s. First, frog IL-10 contained conserved amino acid residues and motifs that are essential for the bioactivity of IL-10, for example, the isoleucine residues at positon 87 (I^87^), which has been proved to be necessary for immunostimulatory function of human IL-10. Substitution of isoleucine with alanine can abrogate the immunostimulatory activity of IL-10 on thymocytes, mast cells, and alloantigenic responses while preserving immunosuppressive activity is concerned with the inhibition of IFN-*γ* production and the prolongation of cardiac allograft survival [30]. This residue was also found in frog IL-10, suggesting that frog IL-10 might possess similar immunostimulatory functions. Also, the four conserved cysteine residues, known to form two disulphide bonds and to be essential for maintaining the structure and bioactivity of human IL-10 [31], were found in frog IL-10 (Figure 2). Two extra cysteine residues existing in fish IL-10, for example, cys27 and cys32 in zebrafish IL-10, but being nonexistent in IL-10s from higher vertebrates, were absent in frog IL-10, suggesting that these two cysteine residues should be fish specific. An alternative scheme of disulphide bonds was predicted in fish IL-10 by disulphide prediction software, DISULFIND [11], but the comparative modeling analysis of carp IL-10 suggested that the two cysteine residues could not form any significant bonds [27]. Thus, further study is needed to reveal their exact function in fish IL-10.

Secondly, frog IL-10 possessed the conserved 3D structure, similar to that of human IL-10, consisting of six *α*-helices termed A, B, C, D, E, and F (Figure 3(a)). The structural model of frog IL-10 in our study was confirmed to be acceptable based on the results of molecular dynamics analysis (Figure 3(b)). It had been proved that these six *α*-helices in human IL-10 are involved in associating with the other monomer to form two interpenetrating domains and that N-terminus, helix A, AB loop, DE loop, and helix F of human IL-10 were active sites for interacting with its receptors [32, 33]. That the conserved six *α*-helices also existed in frog IL-10 suggested that the monomer of frog IL-10 might be firstly activated to form homodimer so as to bind to its receptors. However, shorter helix A and helix C and longer AB loop and CD loop were observed in frog IL-10. In addition, frog IL-10 possessed the same genomic organization as all reported IL-10s, containing five exons and four introns (Figure 1).

Lowly expression of frog IL-10 gene in liver, heart, and stomach tissues was in agreement with the results of the study on trout IL-10 [15]. The relatively high level of frog IL-10 expression occurred in immune related tissues, for example, intestine and spleen (Figure 4(a)), suggesting that the frog IL-10 plays some roles under basal conditions. After LPS stimulation, the expression of frog IL-10 was upregulated greatly in the tissues, including liver, spleen, kidney, intestine, and stomach (Figure 4(b)), indicating that frog IL-10 should act an important role for resistance against bacterial infections. The upregulation of frog IL-10 expression in intestine and stomach also suggested the IL-10 might be involved in mucosal immune response in amphibian [34].

The number of IL genes and their types in the IL-10 loci from various species were different, for example, four ILs named as IL-10, -19, -20, and -24 in mammalian IL-10 locus, IL-10 and -19 in chicken IL-10 locus, and IL-10 and IL-20L in fish IL-10 locus (Figure 5). In the present study, we identified two interleukins in frog IL-10 locus, that is, IL-10 and the homologue of IL-20/19. This homologue identified newly contained six conserved cysteine residues, which were found in mammalian IL-19 and -20 and fish IL-20L [11, 35]. On the contrary, there were four cysteine residues in most of IL-10s and two in human IL-24 (Figure 2). Sequence identities also supported that this novel IL was the homologue of mammalian IL-19/20. Its identities of 37.9% and 35.7%, respectively, with human IL-20 and zebrafish IL-20L were slightly higher than the values of identity with frog IL-10 and other IL-10 family members, ranging from 23.9% to 32.4% (Table 2). The finding of IL-19/20 homologue in frog suggested that the divergence of IL-19 and IL-20 might occur after amphibian appearance during biological evolution. Of course, the function and the potential receptors of the homologue should be further studied to reveal its exact evolutionary position.

## Figures and Tables

**Table 1:** Primers used in the study.

Primer	Sequence (from 5′ to 3′)	Application
UPM	CTAATACGACTCACTATAGGGC	RACE-PCR
IL10-5Rout	CATCTCCGCTTTGACATTTCACCGT	5′-RACE
IL10-5Rin	CCGTTTTGCATGTGAAGAAGA	5′-RACE
IL10-3Fout	AGCAAGGTATCTACAAGGCAATGGG	3′-RACE
IL10-3Fin	ATGGGAGAATTCGATATTTTGATTG	3′-RACE
xeIL-10F1	TGGAAATTGTCTTACTTCAA	Real-time PCR
xeIL-10R1	TGTTTAATTTGCTTGATAGC	Real-time PCR
*β*-Actin-F	GGTCGCCCAAGACATCAG	Real-time PCR
*β*-Actin-R	GCATACAGGGACAACACA	Real-time PCR

**Table 2:** Sequence identities among frog IL-10, frog IL-20, and IL-10 family members from other vertebrates.

	Frog IL-10	Frog IL-20
Frog IL-10	—	
Frog IL-20	29.1	—
Human IL-10	56.5	28.2
Human IL-19	25.5	32.4
Human IL-20	35.5	37.9
Human IL-24	18.3	25.4
Chicken IL-10	58.3	29.1
Chicken IL-19	32.2	35.5
Zebrafish IL-10	41.7	23.9
Zebrafish IL-20L	35.7	35.7
